# Mental health and help-seeking behaviors among Mozambican youth: insights from a post-pandemic National Survey Amidst Internal Conflict

**DOI:** 10.1007/s00127-025-02817-3

**Published:** 2025-01-23

**Authors:** Omid Dadras

**Affiliations:** Research Center for Child Psychiatry, University of Turku, Turku, Finland

**Keywords:** Mental health, Mozambican youth, Depression, Anxiety, Help-seeking behavior

## Abstract

**Purpose:**

This study aimed to investigate the prevalence and sociodemographic determinants of major depressive disorder (MDD) and generalized anxiety disorder (GAD) among Mozambican youth aged 15–24 years, as well as their help-seeking behaviors.

**Methods:**

Data from 8,154 youth participants in the 2022–23 Mozambique Demographic Health Survey were analyzed. MDD and GAD were assessed using the PHQ-9 and GAD-7 scales, respectively. Univariate and multivariate logistic regression analyses were conducted to examine associations between sociodemographic factors and mental health outcomes and health-seeking behaviors**.**

**Results:**

The prevalence of MDD and GAD among Mozambican youth was 7.5% for each condition. However, only 9.3% of those with either MDD or GAD sought help for their symptoms, primarily from family and friends. Females had significantly higher odds of experiencing both MDD and GAD but lower help-seeking behavior as compared to males. Unemployment, lower household wealth, and being single were associated with higher odds of both disorders and lower odds of help-seeking behaviors. Higher education increased the odds of GAD and help-seeking behaviors. Significant regional variations were observed, with conflict-affected regions including Cabo Delgado, Nampula, and Zambezia showing the highest prevalence of MDD and GAD.

**Conclusion:**

This study reveals substantial mental health challenges among Mozambican youth, with notable disparities across sociodemographic groups and regions. The low rates of help-seeking behavior underscore the need for targeted interventions to improve mental health awareness and access to services for socio-demographically vulnerable youth.

## Introduction

Mental health issues among youth, particularly depression and anxiety, are increasingly prevalent globally and have significant personal, social, and economic implications [9, 20]. These conditions can hinder academic performance, social relationships, and future employment prospects [28]. Depression in adolescents is associated with an increased risk of other mental health disorders and suicidal behaviors [9]. Mental disorders account for a substantial portion of global disability and impose a significant economic burden due to lost productivity and associated health conditions [32]. Prevention and intervention during adolescence are crucial for ensuring healthy transitions to adulthood and improving overall well-being [28]. Addressing these issues requires a comprehensive understanding of the social determinants of mental health. Social determinants play a fundamental role in shaping mental health outcomes, particularly in low- and middle-income countries (LMICs) where socioeconomic factors, social support, and healthcare access influence the prevalence and impact of mental health disorders. Lund et al.’s [27] systematic review in The Lancet Psychiatry [27] highlights that poverty, inequality, and exposure to violence are strongly associated with common mental disorders, especially in LMICs. This framework underscores the importance of understanding how socioeconomic and structural factors contribute to mental health disparities and supports the need for targeted interventions that address both mental health and its social determinants in vulnerable populations, such as Mozambican youth.

Recent studies have highlighted the increasing prevalence of common mental disorders (CMDs), including depression and anxiety, in low- and middle-income countries (LMICs), including Mozambique [24, 29, 36]. In Mozambique, a country facing numerous socio-economic challenges and internal conflict, understanding the social determinants of these mental health issues among youth is crucial for developing effective interventions [2, 43]. Studies have found high rates of depression, anxiety, and other disorders in adolescents and young adults in Mozambique. One study in central Mozambique, including Manica and Sofala provinces, reported that 19% of respondents older than 18 years experienced depressive symptoms and 17% had suicidal ideation in 2016–17 [19]. Another study in primary care settings in Sofala Province reported a prevalence of 31.7% % for at least one CMD among youth aged 15–24 years [36]. Another recent study in Cabo Delgado among internally displaced youth aged 14–20 years also reported high CMD prevalence, with rates of MDD and GAD similar to those found in our study sample of Mozambican youth [29]. The Lovero et al. study, which focuses specifically on youth, showed a high prevalence of internalizing and externalizing disorders in adolescents across Mozambique, further underscoring the critical mental health needs of this age group [26]. This aligns with global trends, as the World Health Organization reported a 25% increase in anxiety and depression worldwide during the first year of the COVID-19 pandemic [54]. Despite the increasingly high prevalence of CMDs, cultural beliefs and stigma surrounding mental health often prevent individuals from seeking help, further complicating the situation [43], with only 26% of those reporting depressive symptoms or suicidal ideation seeking care in central Mozambique study [19].

Previous research has highlighted various factors influencing mental health and care access, including socioeconomic status, education, and social support [33, 46], the impact of global events such as the COVID-19 pandemic [13, 18], and internal conflict [29]. However, there is a lack of comprehensive studies focusing specifically on the youth population in Mozambique, particularly after the pandemic and recent escalating internal conflict in Northern regions. This knowledge gap is particularly concerning given mental health disorders affect 10–20% of children and adolescents globally, with 50% of conditions starting by age 14 and 75% by age 24, with the situation likely exacerbated by the COVID-19 pandemic [7] and exposure to violence [29]. Additionally, the burden of mental health issues in African countries, including Mozambique, has been increasing in recent years, particularly among the young population. This trend can be attributed to several factors, including the predominantly young demographics, high prevalence of common mental disorders, specific vulnerabilities of adolescents, limited mental health resources, and psychosocial distress among in-school adolescents [4, 40]. According to recent reports, there is an extreme shortage of psychiatrists, psychologists, and other mental health practitioners, with services concentrated in urban centers, leaving rural and conflict-affected regions underserved. This shortage creates a substantial barrier to help-seeking, especially for youth, and contributes to low rates of professional intervention [10, 17]. Socioeconomic factors, stigma, lack of awareness, and exposure to stressors such as violence and political instability may further exacerbate mental health problems among the young population [43] and the rapidly growing adolescent population underscores the urgent need for mental health research and intervention [48].

Against this background, this study aims to address this knowledge gap by investigating the prevalence and social determinants of depression and generalized anxiety and help-seeking behaviors among youth aged 15–24 years in Mozambique, using data from a population-based survey conducted in 2022–23. By focusing on this age group, we target a critical period in life when mental health issues often first emerge and can have long-lasting impacts on future development and well-being. Providing a comprehensive understanding of sociodemographic drivers of mental health and help-seeking behaviors in Mozambique, this study aims to inform policy decisions, improve resource allocation, and ultimately enhance mental health outcomes for young people in the country.

## Methods

### Study setting

The Demographic Health Survey 2022–23 in Mozambique (DHS 2022–23) was conducted by the National Statistics Institute and funded by the Government of Mozambique, with support from the United States Agency for International Development (USAID), World Bank, United Nations Children's Fund (UNICEF), Foreign, Commonwealth & Development Office (FCDO), Canadian High Commission, and Gavi. The DHS Program, funded by USAID, provided technical assistance for the survey, which aims to update demographic and health indicators in Mozambique. These data are crucial for monitoring public policy implementation and relevant Sustainable Development Goal (SDG) indicators.

### Sampling and data collection

The sample design for the Mozambique DHS 2022–23 was structured in two stages to provide national-level estimates, as well as estimates for urban and rural areas across Mozambique's ten provinces (Niassa, Cabo Delgado, Nampula, Zambézia, Tete, Manica, Sofala, Inhambane, Gaza, and Maputo), including the City of Maputo, which holds provincial status as the capital. In the first stage, conglomerates (clusters) were selected, consisting of enumeration areas (AEs) delineated based on the 2017 IV General Population Census and Housing (IV RGPH). A total of 619 enumeration areas were chosen using probability proportional to size, where the size was determined by the number of households in each defined layer. Of these, 232 were from urban areas and 387 from rural areas. Due to security concerns, eight districts in Cabo Delgado province (Ibo, Macomia, Mocímboa da Praia, Mueda, Muidumbe, Nangade, Palma, and Quissanga) were excluded from the sample selection. In the second stage, 26 households were systematically selected with equal probability within each enumeration area. This methodology resulted in 16,045 households being identified for inclusion in the IDS 2022–23. The final count of households was slightly less than planned, specifically 16,094, due to incomplete surveys in two selected areas—one in Cabo Delgado and another in Zambézia Province, both rural—owing to security reasons. The survey included all women aged 15–49 who were regular residents or overnight visitors the night before the in-home interviews, totaling 13,976 eligible women. Of these, interviews were completed with 13,183 women, yielding a response rate of 94%. Similarly, all men aged 15–54, regardless of their residence status, were eligible for interview. A total of 6,282 eligible men were identified, and 5,380 were successfully interviewed, resulting in an 86% response rate. For the purpose of the present study, we only included 8154 youth aged 15–24 years old in the analysis.

### Data collection

The Mozambique DHS 2022–23 utilized five main questionnaires: household questionnaire, questionnaire for women aged 15–49, questionnaire for men aged 15–54, biomarker questionnaire, and water quality test questionnaire. These questionnaires were developed from the DHS program model questionnaires and were translated into Portuguese. They were further adapted to address specific population and health issues pertinent to Mozambique. Trained field workers conducted the interview. Due to the COVID-19 pandemic coinciding with the preparation of IDS 2022–23, protocols for COVID-19 mitigation were integrated into the planning process. These protocols aimed to prevent infection among all personnel involved in the data collection process. Additionally, contingency measures were established to minimize the impact in case of infection.

### Study variables

#### Mental health outcome

*Major Depressive disorder (MDD)* was evaluated using the Patient Health Questionnaire (PHQ-9) in Mozambique DHS 2022–23. This is a 9-item tool that assessed respondents' experiences of nine common depressive symptoms over the past two weeks. The PHQ-9 employs a 4-point rating scale from 0 ("not at all") to 3 ("always") (Kroenke & Spitzer, 2002). This questionnaire was translated and validated for the Mozambican population, demonstrating good internal consistency with a Cronbach’s alpha of 0.84 and an area under the receiver operating characteristic curve (AUROC) of 0.81 (95% CI: 0.73, 0.89) [16]. A cutoff score of ≥ 10 indicates moderate to severe depression symptoms [16],Kroenke & Spitzer, 2002) and coded as “1 = yes” for MDD. For the PHQ-9, a cut-off score of ≥ 10 was used to indicate moderate to severe depression, aligning with validation studies in Sub-Saharan Africa that demonstrate acceptable sensitivity and specificity at this level, while managing false positives in populations where mental health resources are limited [6, 11, 14, 42]. While the Cumbe et al. study for the Mozambican adaptation (PHQ-9-MZ) suggests various cut-off scores (e.g., ≥ 9) depending on programmatic priorities, the ≥ 10 cut-off has been widely validated across LMICs and allows for a more focused identification of individuals with potentially moderate to severe symptoms in resource-limited settings [14, 35].

*Generalized Anxiety Disorder (GAD)* was measured using the seven-item GAD-7 scale, with scores of ≥ 10 indicating moderate to severe GAD [49] which was coded as “1 = yes” for GAD. For the Mozambican population, the GAD-7 showed good internal consistency with a Cronbach's alpha of 0.84, demonstrating good sensitivity and specificity (both > 0.70) [26]. For the GAD-7, a cut-off score of ≥ 10 was selected to detect moderate or higher anxiety levels, consistent with international validation studies and supported by findings from the Lovero et al. study in Mozambique [26]. Although Lovero et al. found lower sensitivity at the ≥ 10 cut-off in adolescent populations, they noted that higher cut-offs might help identify individuals at risk in resource-limited contexts where managing high sensitivity and specificity is crucial. Lovero et al. also emphasize that the selection of a higher cut-off should consider screening objectives, disorder prevalence, and available treatment resources [26]. Therefore, we retained the ≥ 10 cut-off for our study to identify those with moderate to severe anxiety, aligning with both local recommendations and broader LMIC validation [1, 11, 21, 30].

### Help-seeking behaviors

*Ever tried to seek help for the things you experienced*, a binary variable with alternative responses of “no = 0” and “yes = 1”, which was followed by the question about the person whom the participant sought help from, with alternative responses of “No help = 0”, “Doctor/Medical professional = 1”, “Social worker/Community health worker = 3″, “Religious leader = 4″, and” Family/Friends = 5”.

### Sociodemographic covariates

Age groups (15–19 = 0, 20–24 = 1), sex (male = 0, female = 1), education (no education = 0, primary = 1, secondary/higher = 2), marital status (never married = 1, married/cohabited = 2, widows/separated/divorced = 3), currently working (indicates any form of employment, including formal and informal work. However, the survey did not further categorize employment by type, such as full-time versus part-time, or formal versus informal work. The responses were no = 0, yes = 1), wealth index (In DHS surveys, the wealth index is calculated using principal component analysis (PCA) of household assets to rank participants into relative wealth quintiles, accounting for factors such as urban/rural residence and household assets. This method reflects relative socioeconomic status within the population [52]. It was coded as poor = 0, middle = 2, rich = 3), religion (Christian = 0, Islam = 1, Zion = 2, Evangelical/Pentecostal = 3, no religion = 4, others = 5), place of living (urban = 0, rural = 1), region (Cidade de Maputo = 0, Niassa = 1, Cabo Delgado = 2, Nampula = 3, Zambezia = 4, Tete = 5, Manica = 6, Sofala = 7, Inhambane = 8, Gaza = 9, Maputo = 10).

### Statistical analysis

Descriptive statistics were used to describe the distribution of sociodemographic characteristics and prevalence of MDD, GAD, and help-seeking behavior among youth aged 15–24 in Mozambique DHS 2022–23. Univariate and multivariable logistic regression analyses were employed to estimate the odds of MDD, GAD, and help-seeking behavior across sociodemographic factors. Variables with a p-value greater than 0.2 in bivariate analysis were excluded from the multivariable models. In each final multivariable model, we adjusted for key sociodemographic covariates, including age, sex, education, marital status, employment status, wealth index, religion, urban/rural residence, and region. These covariates were selected based on both theoretical relevance and statistical significance criteria to ensure a comprehensive model fit. To address potential collinearity, we assessed multicollinearity using variance inflation factors (VIF) and the “collin” command in Stata. Results were reported as odds ratios (ORs) and 95% confidence intervals (CIs). The distribution of the person from whom the respondent sought help was presented as a stacked bar chart. Missing data were treated in a listwise manner. To ensure that the results were nationally representative, sampling weights were calculated based on probability proportional to size (PPS) at each sampling stage, accounting for factors such as urban/rural distribution, response rates, and regional representation. These weights adjust for variations in sampling probability across different regions and ensure that the sample accurately reflects Mozambique’s demographic structure. Weights were further adjusted for response rates within each stratum (urban/rural) and across regions to address any potential non-response bias. In our analysis, we applied these sampling weights and used complex survey design procedures in STATA 18 (StataCorp LLC, College Station, TX, USA) to accurately account for clustering and stratification, ensuring that estimates are representative of the national youth population. A p-value of < 0.05 was considered statistically significant.

## Results

### Sociodemographic characteristics

The study sample consisted of 8,154 youths aged 15–24 years from the Mozambique DHS 2022–23. The distribution of the sample by sociodemographic characteristics is presented in Table 1. The majority of the respondents were aged 15–19 years (54.7%), female (70.8%), and had at least primary education (86.1%). More than half of the respondents (54.0%) were never married, while 40.6% were married or cohabitating. The majority were not currently working (66.9%), and nearly half belonged to the rich wealth index category (47.8%). Most respondents identified as Catholic (29.4%) or Evangelical/Pentecostal (27.4%), lived in rural areas (59.6%), and were predominantly from the Nampula region (24.0%).

**Table 1:** Sociodemographic characteristics of youth aged 15–24 years in Mozambique DHS 2022–23 (n = 8154)

Age	N (weighted%)
15–19	4548 (54.7)
20–24	3606 (45.3)
Sex	
Male	2420 (29.2)
Female	5734 (70.8)
Education	
No education	940 (13.9)
Primary	3307 (44.2)
Secondary/higher	3907 (41.9)
Marital status	
Never married	4638 (54.0)
Married/cohabited	3088 (40.6)
Widows/separated/divorced	428 (5.4)
Currently working	
No	5221 (66.9)
Yes	2933 (33.1)
Wealth index *	
Poor	2188 (35.1)
Middle	1450 (17.1)
Rich	4516 (47.8)
Religion	
Catholic	1866 (29.4)
Islamic	1517 (21.0)
Zion	1106 (10.6)
Evangelical/Pentecostal	2640 (27.4)
No religion	800 (9.1)
Others	225 (2.5)
Place of living	
Urban	3590 (40.4)
Rural	4564 (59.6)
Region	
Niassa	711 (6.7)
Cabo Delgado	795 (5.2)
Nampula	914 (24.0)
Zambezia	638 (16.8)
Tete	709 (9.8)
Manica	779 (7.1)
Sofala	823 (7.4)
Inhambane	580 (3.8)
Gaza	770 (5.0)
Maputo	717 (9.4)
Cidade de Maputo	718 (4.7)

^*^ The distribution here reflects the socioeconomic status within the study sample of youth aged 15–24 years, which may differ from the national distribution for the broader population

### Prevalence and Association of MDD with Sociodemographic Characteristics among Mozambican Youth

As Table 2 presents, the overall prevalence of MDD in the sample was 7.5%. Youth aged 20–24 had a higher prevalence of MDD (8.5%) compared to those aged 15–19 (6.6%). Univariate analysis showed that older youth had 1.32 times the odds of experiencing MDD compared to younger youth, but this association was not significant after adjustment (AOR = 1.22, 95% CI: 0.91, 1.62). Females had a significantly higher prevalence of MDD (9.8%) compared to males (2.7%). Females had over six times the odds of experiencing MDD compared to males in both unadjusted (OR = 6.19, 95% CI: 3.96, 9.69) and adjusted models (AOR = 5.85, 95% CI: 3.50, 9.78). Youth with primary education had a higher prevalence of MDD (9.2%) compared to those with no education (7.5%) or secondary/higher education (5.7%). Education level did not show significant associations with MDD in neither univariate or multivariable analyses. Widowed/separated/divorced youth had higher prevalence (13.6%) compared to married or cohabiting youth (9.1%) and never married (5.7%) and Being widowed/separated/divorced was significantly associated with higher odds of MDD in the adjusted model (AOR = 1.76, 95% CI: 1.02, 2.31). Youth not currently working had a higher prevalence of MDD (9.5%) compared to those who were employed (3.5%). Employment was associated with lower odds of MDD in unadjusted analysis (OR = 0.34, 95% CI: 0.25, 0.46), but this association was not significant after adjustment. Youth from poorer households had a higher prevalence of MDD (9.2%) compared to those from middle (6.4%) and rich households (6.6%). Although a higher wealth index (middle and rich) was associated with lower odds of MDD, this association attenuated and became insignificant in the adjusted model. Youth practicing Islam had a higher prevalence of MDD (11.2%) compared to other religious groups, with the lowest prevalence among the Zion (2.9%). Although in univariate analyses, Zion, Evangelical/Pentecostal and having no religion were associated with lower odds of MDD (Table 2), none of the religious affiliations showed significant associations with MDD after adjustment. The prevalence of MDD was similar in urban (7.7%) and rural (7.3%) areas. Place of living was not significantly associated with MDD in the either unadjusted or adjusted models. There was significant regional variation in MDD prevalence, with the highest in Nampula (16.2%) and the lowest in Gaza (0.8%). Even after adjustment for other sociodemographic covariates, several regions showed significantly higher odds of MDD compared to Cidade de Maputo, including Cabo Delgado (AOR = 6.01, 95% CI: 2.57, 14.02), Nampula (AOR = 10.87, 95% CI: 5.14, 23.00), Zambezia (AOR = 5.31, 95% CI: 2.47, 11.43), and Sofala (AOR = 3.59, 95% CI: 1.87, 6.90).

**Table 2:** The prevalence and association of MDD with different sociodemographic characteristics among Mozambican youth

	MDD		
	N (%)	OR (95%CI)	AOR (95%CI)
Total sample (n = 8152)	7.5		
*Age*			
15–19	6.6	Reference	Reference
20–24	8.5	1.32 (1.03, 1.69)*	1.22 (0.91, 1.62)
*Sex*			
Male	2.7	Reference	Reference
Female	9.8	6.19 (3.96, 9.69)*	5.85 (3.50, 9.78)*
*Education*			
No education	7.5	Reference	Reference
Primary	9.2	1.23 (0.84, 1.81)	1.41 (0.91, 2.18)
Secondary/higher	5.7	0.75 (0.50, 1.12)	1.29 (0.72, 2.31)
*Marital status*			
Never married	5.7	Reference	Reference
Married/cohabited	9.1	1.67 (1.25, 2.23)*	1.02 (0.73, 1.43)
Widows/separated/divorced	13.6	2.64 (1.64, 4.24)*	1.76 (1.02, 2.31)*
*Currently working*			
No	9.5	Reference	Reference
Yes	3.5	0.34 (0.25, 0.46)*	0.80 (0.57, 1.12)
*Wealth index*			
Poor	9.2	Reference	Reference
Middle	6.4	0.68 (0.47, 0.97)*	0.98 (0.66, 1.43)
Rich	6.6	0.70 (0.50, 0.98)*	1.11 (0.69, 1.78)
*Religion*			
Catholic	9.9	Reference	Reference
Islamic	11.2	1.14 (0.81, 1.61)	0.93 (0.66, 1.32)
Zion	2.9	0.27 (0.17, 0.43)*	0.77 (0.42, 1.42)
Evangelical/Pentecostal	5.1	0.49 (0.34, 0.71)*	1.17 (0.72, 1.89)
No religion	4.1	0.39 (0.21, 0.72)*	1.07 (0.53, 2.17)
Others	4.8	0.46 (0.21, 1.02)	1.29 (0.54, 3.03)
*Place of living*			
Urban	7.7	Reference	Reference
Rural	7.3	0.95 (0.68, 1.32)	0.82 (0.53, 1.27)
*Region*			
Cidade de Maputo	2.2	Reference	Reference
Niassa	2.8	1.25 (0.64, 2.44)	1.52 (0.70, 3.31)
Cabo Delgado	9.6	4.65 (2.31, 9.37)*	6.01 (2.57, 14.02)*
Nampula	16.2	8.45 (4.61, 15.50)*	10.87 (5.14, 23.00)*
Zambezia	9.3	4.50 (2.23, 9.07)*	5.31 (2.47, 11.43)*
Tete	2.4	1.06 (0.48, 2.34)	1.34 (0.57, 3.17)
Manica	1.2	0.53 (0.22, 1.27)	0.59 (0.23, 1.53)
Sofala	6.8	3.18 (1.72, 5.89)*	3.59 (1.87, 6.90)*
Inhambane	1.1	0.49 (0.19, 1.27)	0.58 (0.22, 1.55)
Gaza	0.8	0.34 (0.13, 0.86)*	0.37 (0.14, 0.98)*
Maputo	3.8	1.73 (0.79, 3.76)	1.82 (0.82, 4.02)

* p < 0.05

### Prevalence and Association of GAD with Sociodemographic Characteristics among Mozambican Youth

As Table 3 outlines, the overall prevalence of GAD was 7.5%. Youth aged 20–24 had a higher prevalence of GAD (8.9%) compared to those aged 15–19 (6.3%). The univariate analysis indicated higher odds for the older age group (OR = 1.44, 95% CI: 1.13, 1.83), but this was not significant in the adjusted model (AOR = 1.22, 95% CI: 0.93, 1.60). Females had a significantly higher prevalence of GAD (9.9%) compared to males (1.6%). Females had nearly seven times the odds of experiencing GAD compared to males in both unadjusted (OR = 6.90, 95% CI: 4.54, 10.48) and adjusted models (AOR = 6.69, 95% CI: 4.16, 10.77). Youth with primary education had a higher prevalence of GAD (9.1%) compared to those with no education (7.0%) or secondary/higher education (5.9%). Although not significant in univariate analysis, after adjustment, primary education (AOR = 1.58, 95% CI: 1.02, 2.44) and secondary/higher education (AOR = 1.65, 95% CI: 1.00, 2.75) were significantly associated with GAD. Widowed/separated/divorced youth had a higher prevalence (13.7%) compared to never married (5.1%) and married or cohabiting youth (9.8%). Being widowed/separated/divorced was significantly associated with higher odds of GAD in both the unadjusted (OR = 2.99, 95% CI: 1.83, 4.86) and the adjusted model (AOR = 2.01, 95% CI: 1.17, 3.45). Youth not currently working had a higher prevalence of GAD (9.4%) compared to those who were employed (3.5%). Employment was associated with lower odds of GAD in unadjusted analysis (OR = 0.35, 95% CI: 0.26, 0.46), but this association was not significant after adjustment. Youth from poor households had a higher prevalence of GAD (9.7%) compared to those from middle (5.8%) and rich households (6.5%). No significant associations were found between the wealth index and GAD in the adjusted model, even though it was significant in univariate analyses. Youth practicing Islam had a higher prevalence of GAD (9.3%) compared to other religious groups, with the lowest prevalence among the Zion (3.0%). None of the religious affiliations showed significant associations with GAD after adjustment, even though in univariate analyses, Zion, Evangelical/Pentecostal, and having no religion were associated with lower odds of GAD. The prevalence of GAD was similar in urban (7.3%) and rural (7.6%) areas. Place of living was not significantly associated with GAD in both unadjusted and adjusted models. There was significant regional variation in GAD prevalence, with the highest in Nampula (17.9%) and the lowest in Gaza (1.4%). Several regions showed significantly higher odds of GAD compared to Cidade de Maputo even after adjustment for other sociodemographic covariates. These include Cabo Delgado (AOR = 2.38, 95% CI: 1.01, 5.80), Nampula (AOR = 7.49, 95% CI: 3.88, 14.43), Zambezia (AOR = 2.01, 95% CI: 1.07, 3.75), Sofala (AOR = 1.88, 95% CI: 1.03, 3.42), and Inhambane (AOR = 0.19, 95% CI: 0.05, 0.67).

**Table 3:** The prevalence and association of GAD with different sociodemographic characteristics among Mozambican youth

	GAD		
	N (%)	OR (95%CI)	AOR (95%CI)
Total sample (n = 8151)	7.5		
*Age*			
15–19	6.3	Reference	Reference
20–24	8.9	1.44 (1.13, 1.83)*	1.22 (0.93, 1.60)
*Sex*			
Male	1.6	Reference	Reference
Female	9.9	6.90 (4.54, 10.48)*	6.69 (4.16, 10.77)*
*Education*			
No education	7.0	Reference	Reference
Primary	9.1	1.33 (0.91, 1.95)	1.58 (1.02, 2.44)*
Secondary/higher	5.9	0.83 (0.57, 1.21)	1.65 (1.00, 2.75)*
*Marital status*			
Never married	5.1	Reference	Reference
Married/cohabited	9.8	2.05 (1.50, 2.81)*	1.28 (0.90, 1.82)
Widows/separated/divorced	13.7	2.99 (1.83, 4.86)*	2.01 (1.17, 3.45)*
*Currently working*			
No	9.4	Reference	Reference
Yes	3.5	0.35 (0.26, 0.46)*	0.84 (0.62, 1.15)
*Wealth index*			
Poor	9.7	Reference	Reference
Middle	5.8	0.57 (0.39, 0.84)*	0.78 (0.52, 1.16)
Rich	6.5	0.65 (0.49, 0.86)*	0.96 (0.62, 1.50)
*Religion*			
Catholic	9.3	Reference	Reference
Islamic	11.6	1.28 (0.91, 1.80)	1.05 (0.73, 1.51)
Zion	3.0	0.30 (0.18, 0.49)*	0.69 (0.37, 1.29)
Evangelical/Pentecostal	5.2	0.53 (0.37, 0.76)*	1.23 (0.78, 1.94)
No religion	4.8	0.49 (0.28, 0.87)*	1.39 (0.72, 2.67)
Others	5.3	0.54 (0.25, 1.20)	1.36 (0.57, 3.12)
*Place of living*			
Urban	7.3	Reference	Reference
Rural	7.6	1.04 (0.78, 1.40)	0.95 (0.62, 1.44)
*Region*			
Cidade de Maputo	3.2	Reference	Reference
Niassa	2.1	0.66 (0.34, 1.30)	0.71 (0.32, 1.57)
Cabo Delgado	6.7	2.18 (1.08, 4.40)*	2.38 (1.01, 5.80)*
Nampula	17.9	6.62 (4.06, 10.82)*	7.49 (3.88, 14.43)*
Zambezia	6.2	1.99 (1.15, 3.45)*	2.01 (1.07, 3.75)*
Tete	2.9	0.92 (0.51, 1.67)	0.99 (0.52, 1.90)
Manica	3.1	0.96 (0.54, 1.72)	0.96 (0.49, 1.88)
Sofala	5.9	1.89 (1.10, 3.26)*	1.88 (1.03, 3.42)*
Inhambane	0.6	0.17 (0.05, 0.59)*	0.19 (0.05, 0.67)*
Gaza	1.4	0.45 (0.21, 0.95)*	0.46 (0.21, 1.00)*
Maputo	4.8	1.53 (0.81, 2.89)	1.54 (0.80, 2.99)

* p < 0.05

### Prevalence and Association of Help-Seeking Behavior with Sociodemographic Characteristics among Mozambican Youth

As Table 4 illustrates, the overall prevalence of help-seeking was 9.3%. Youth aged 20–24 had a slightly higher prevalence of help-seeking behavior (10%) compared to those aged 15–19 (8.6%); however, it was not significant in either unadjusted or adjusted models. Males had a significantly higher prevalence of help-seeking behavior (17.4%) compared to females (5.9%). Males had significantly higher odds of seeking help compared to females in both unadjusted (OR = 0.30, 95% CI: 0.24, 0.38) and adjusted models (AOR = 0.44, 95% CI: 0.34, 0.58). Youth with secondary/higher education had the highest prevalence of help-seeking behavior (13.7%) compared to those with primary (6.0%) and no education (4.1%). Youth with secondary/higher education were significantly more likely to seek help compared to those with no education in both unadjusted (OR = 3.70, 95% CI: 2.35, 5.81) and adjusted models (AOR = 1.52, 95% CI: 1.01, 2.30). Never-married youth had a higher prevalence (11.4%) compared to widowed/separated/divorced youth (10.8%) and married or cohabiting youth (6.4%). Although in univariate analysis, the odds of seeking help were lower in married/cohabited youth as compared to never-married (OR = 0.53, 95% CI: 0.42, 0.67), the multivariable analysis indicated higher odds of seeking help in widowed/separated/divorced youth (AOR = 1.32, 95% CI: 1.01, 2.30). Youth currently working had a higher prevalence of help-seeking behavior (16.2%) compared to those not working (5.7%). Employed youth had significantly higher odds of seeking help compared to those not working in both unadjusted (OR = 3.17, 95% CI: 2.56, 3.92) and adjusted models (AOR = 1.94, 95% CI: 1.52, 2.47). Youth from rich households had a higher prevalence of help-seeking behavior (13.2%) compared to those from middle (7.2%) and poor households (4.0%). Although in the unadjusted model, both youths from rich and middle households had significantly higher odds of seeking help compared to those from poor households, in the adjusted model this remained significant only for those from rich households (AOR = 1.75, 95% CI: 1.12, 2.73). Youth-practicing Catholics had a lower prevalence of help-seeking behavior (6.1%) compared to other religious groups, with the highest prevalence among the Evangelical/Pentecostal (12.8%). Contrary to univariate analysis where the odds of seeking help were higher in Zion, Evangelical/Pentecostal, no religion group; none of the religious affiliations showed significant associations with help-seeking behavior after adjustment. Youth from urban areas had a higher prevalence of help-seeking behavior (12.5%) compared to those from rural areas (6.7%). Despite lower odds of seeking help in rural youth in the unadjusted model, no significant association was observed in the adjusted model. There was significant regional variation in help-seeking behavior, with the highest in Gaza (20.9%) and the lowest in Nampula (1.8%). Youth from Gaza, Maputo, Sofala, Tete, and Niassa had significantly higher odds of seeking help compared to those from Cidade de Maputo in both unadjusted and adjusted models (Table 4).

**Table 4:** The prevalence and association of help-seeking behavior with different sociodemographic characteristics among Mozambican youth

	Sought help for their symptoms		
	N (%)	OR (95%CI)	AOR (95%CI)
Total sample (n = 5213) ^a^	9.3		
*Age*			
15–19	8.6	Reference	Reference
20–24	10	1.18 (0.97, 1.45)	1.17 (0.89, 1.54)
*Sex*			
Male	17.4	Reference	Reference
Female	5.9	0.30 (0.24, 0.38)*	0.44 (0.34, 0.58)*
*Education*			
No education	4.1	Reference	Reference
Primary	6.0	1.49 (0.96, 2.31)	1.05 (0.71, 1.56)
Secondary/higher	13.7	3.70 (2.35, 5.81)*	1.52 (1.01, 2.30)*
*Marital status*			
Never married	11.4	Reference	Reference
Married/cohabited	6.4	0.53 (0.42, 0.67)*	0.97 (0.73, 1.29)
Widows/separated/divorced	10.8	0.95 (0.64, 1.41)	1.32 (1.01, 2.30)*
*Currently working*			
No	5.7	Reference	Reference
Yes	16.2	3.17 (2.56, 3.92)*	1.94 (1.52, 2.47)*
*Wealth index*			
Poor	4.0	Reference	Reference
Middle	7.2	1.85 (1.19, 2.88)*	1.05 (0.65, 1.71)
Rich	13.2	3.63 (2.52, 5.24)*	1.75 (1.12, 2.73)*
*Religion*			
Catholic	6.1	Reference	Reference
Islamic	7.0	1.15 (0.76, 1.75)	1.10 (0.69, 1.77)
Zion	11.4	1.98 (1.37, 2.87)*	0.96 (0.66, 1.38)
Evangelical/Pentecostal	12.8	2.26 (1.64, 3.11)*	0.94 (0.68, 1.30)
No religion	11.1	1.93 (1.21, 3.07)*	0.78 (0.48, 1.28)
Others	11.9	2.08 (1.05, 4.12)*	0.80 (0.38, 1.72)
*Place of living*			
Urban	12.5	Reference	Reference
Rural	6.7	0.51 (0.40, 0.64)*	0.93 (0.72, 1,21)
*Region*			
Cidade de Maputo	9.6	Reference	Reference
Niassa	2.2	2.68 (1.79, 4.00)*	3.86 (2.24, 6.67)*
Cabo Delgado	7.4	0.75 (0.40, 1.40)	1.26 (0.58, 2.71)
Nampula	1.8	0.17 (0.08, 0.34)*	0.32 (0.16, 0.68)*
Zambezia	2.5	0.24 (0.12, 0.50)*	0.46 (0.20, 1.03)
Tete	11.4	1.21 (0.69, 2.13)	2.05 (1.12, 3.76)*
Manica	9.2	0.95 (0.56, 1.62)	1.64 (0.90, 2.98)
Sofala	18.5	2.14 (1.42, 3.25)*	3.13 (1.97, 4.95)*
Inhambane	12.5	1.35 (0.82, 2.21)	1.79 (1.00, 3.20)*
Gaza	20.9	2.49 (1.69, 3.67)*	3.06 (1.94, 4.81)*
Maputo	16.3	1.83 (1.22, 2.77)*	1.99 (1.24, 3.19)*

^a^ Help-seeking behavior was assessed only among participants who reported symptoms of MDD or GAD, resulting in a reduced sample size for this analysis (n = 5,213). * p-value < 0.05

### Source of help among Mozambican youth

**Fig. 1:**
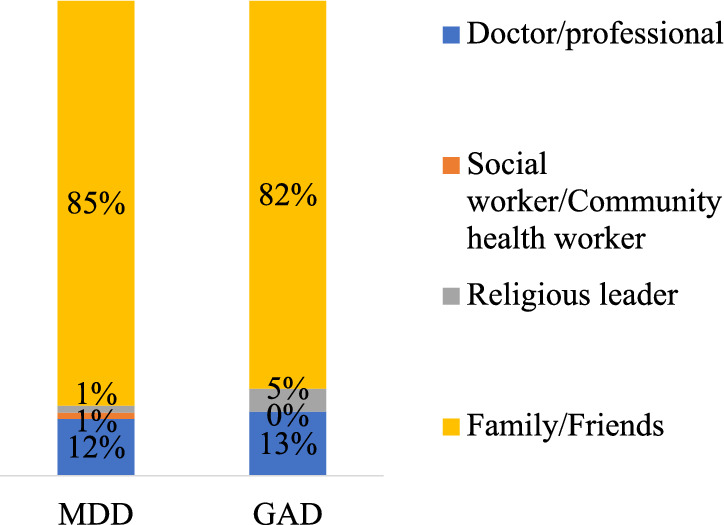
The person from whom the respondent sought help

As shown in Fig. 1, over 80% of respondents with MDD and GAD symptoms sought help from a family member or a friend. Only about 12% visited a health professional, and very few sought consolation from a religious leader.

## Discussion

The findings of this study provide valuable insights into the prevalence and social determinants of MDD and GAD as well as help-seeking behavior among Mozambican youth aged 15–24 years. The overall prevalence of MDD and GAD at 7.5% and the significant regional variations observed warrant careful consideration, especially in the context of Mozambique's ongoing internal conflicts and their consequences. Recent studies in Mozambique reveal significant prevalence rates of common mental disorders among various populations. For instance, research conducted in primary care settings found that approximately 31.7% of patients aged 15–24 met the criteria for at least one mental disorder, which is more than three times the rate observed in our study [6]. This disparity may be attributed to the higher concentration of individuals with poor mental health in primary care settings compared to the general population. Conversely, another study focused on internally displaced populations in Cabo Delgado reported prevalence rates of MDD and GAD similar to those in our study for youth aged 14–20 years [29]. The observed regional disparities in mental health conditions reflect a complex interaction of socioeconomic factors, cultural differences, and notably, the impact of internal conflicts in Mozambique [29, 36]. The northern region of Cabo Delgado, for instance, has been severely affected by an ongoing insurgency since 2017. The resulting widespread displacement and trauma have extended to neighboring provinces like Nampula, Zambezia, and Sofala, where significantly higher rates of MDD and GAD have been reported compared to Cidade de Maputo (the capital province). Factors such as loss of social support networks, economic instability and loss of livelihoods, exposure to violence and trauma, and disruption of healthcare services, including mental health support, have profound implications for the mental health of Mozambican youth [8, 12, 34]. These findings underscore the severe mental health consequences of conflict and displacement, suggesting a need for targeted mental health interventions in these high-risk regions.

Moreover, as documented in our study, socioeconomically vulnerable groups including females, individuals from lower-wealth households, and those who are married or cohabited as well as those widowed, separated, or divorced, are at an elevated risk of mental health issues. Women, particularly the young group, are often more likely to suffer from MDD and GAD but are less likely to seek help due to stigma, cultural norms, and higher exposure to domestic violence [29] as observed in our study. Additionally, young married or cohabited women may face additional stressors related to reproductive health, including early marriage, unintended pregnancies, and limited access to healthcare services, which can increase vulnerability to mental health issues. Moreover, the higher prevalence of MDD and GAD observed among married or cohabited as well as widowed, separated, or divorced youth in this study compared to never-married individuals points to the significant impact of relationship status on mental health. This disparity can be attributed to factors such as loss and grief, social isolation, financial stress, identity and future uncertainty, and societal stigma [23, 50]. Addressing these issues necessitates targeted mental health interventions, including grief counseling, social support groups, financial counseling, and culturally sensitive approaches to reduce stigma and provide comprehensive support for youth experiencing relationship transitions or loss [47]. On the other hand, the lower prevalence of MDD and GAD among employed youth highlights the potential protective effect of employment on mental health. Employment can provide a sense of purpose, financial stability, and social interaction, all of which contribute to better mental health outcomes [41]. This finding underscores the importance of youth employment initiatives as a strategy for improving mental health outcomes, suggesting that policies and programs aimed at increasing job opportunities for young people could play a crucial role in mitigating mental health issues [5]. In the context of Mozambique, particularly in regions like Cabo Delgado and Nampula that are severely affected by conflict and displacement, the loss of employment due to displacement and the limited economic opportunities exacerbate mental health issues among youth [53]. Thus, targeted employment initiatives, with a strong focus on gender equality, can significantly enhance mental health outcomes among vulnerable youth.

Another important finding was the association between higher education and increased prevalence of GAD but not MDD among Mozambican youth. Higher education often brings increased academic pressure, performance expectations, and future career uncertainties, which can contribute to heightened anxiety levels [15, 22]. However, these stressors are often time-limited, which may be more likely to produce anxiety than long-term depressive symptoms [22]. It is important to note that while these factors may explain the association between higher education and GAD, the relationship between education and mental health is complex and can vary based on individual and contextual factors. More specific research in the Mozambican context would be needed to fully understand this relationship. Religious affiliations such as Zion and Evangelical/Pentecostal and non-religious youth were less likely to suffer from MDD and GAD as compared to Christians in univariate analysis; however, this association disappeared in the adjusted model which can be due to the differential distribution of these religions across regions, and higher concentration of Muslims and Christians in Northern regions that affected by internal conflict. The ongoing conflict in northern Mozambique has likely led to increased rates of MDD and GAD among the population in these areas, regardless of religious affiliation.

Our findings indicated lower help-seeking behaviors in these provinces particularly Cabo Delgado, Nampula, and Zambezia compared to Cidade de Maputo which can be due to the lower or disrupted access to such services in these regions, perceived stigma, and lack of social support among the displaced population [37, 51]. Additionally, in the adjusted model, being female and living in Nampula and Zambezia were associated with lower and being secondary or higher educated, currently working, high wealth index, and being widows, separated, or divorced were associated with higher help-seeking behavior. The lower help-seeking behaviors among females is a concerning trend that reflects broader gender inequalities in Mozambique. Women face additional barriers such as limited decision-making power, economic dependence, and cultural norms that may discourage seeking help for mental health issues [55]. Additionally, in many African nations, including Mozambique, mental health issues are still heavily stigmatized and often considered taboo [43]. This cultural stigma can prevent youth, particularly females and young people, from acknowledging mental health problems or seeking professional help. There is also a prevalent belief in Mozambique that mental illness is a consequence of spiritual problems rather than a medical condition [43]. This misconception can lead youth to seek help from traditional healers instead of mental health professionals as documented in our study. The lower help-seeking behaviors in Nampula and Zambezia, despite not being as directly affected by conflict as Cabo Delgado, while having higher MDD and GAD prevalence, suggest that other factors such as cultural norms, economic conditions, and health system capacity play a role in these regional disparities and warrant further investigation. On the other hand, higher levels of education are often associated with increased mental health literacy, awareness of available services, and lower levels of stigma. Therefore, educated individuals may be better equipped to recognize symptoms and understand the importance of seeking help [44]. Additionally, employment can provide both financial means and social connections that facilitate help-seeking. Employed individuals may have better access to mental health services through employee assistance programs or health insurance [31]. Financial resources can also remove barriers to accessing mental health services, such as transportation costs or out-of-pocket expenses for treatment [25]. Higher health-seeking behavior among widowed, separated, or divorced individuals might be due to greater independence in decision-making, reduced stigma in seeking help, and a higher perceived need for support during life transitions [3, 39]. These findings underscore the importance of addressing socioeconomic factors and education in efforts to improve mental health service utilization. Interventions aimed at increasing mental health awareness, reducing stigma, and improving access to services across all socioeconomic groups may be particularly effective in promoting help-seeking behaviors.

Lastly, although not explicitly addressed in the study, the potential impact of the COVID-19 pandemic on mental health outcomes cannot be overlooked. The data collection for this study took place in 2022–23, during the ongoing pandemic, which likely influenced the mental health landscape in Mozambique. The pandemic may have exacerbated existing mental health challenges through economic disruptions and job losses, social isolation due to lockdowns and distancing measures, increased stress and anxiety related to health concerns, disruptions to education, and future prospects for youth [38, 45]. In particular, the pandemic's effects may have disproportionately impacted vulnerable populations identified in the present study including females and those living in the regions affected by internal conflict, potentially contributing to the observed disparities in mental health outcomes.

### Limitations

This study has several limitations and strengths. This study’s cross-sectional design limits causal inferences. While associations between social determinants and mental health outcomes are identified, we cannot establish the direction of causation, raising the potential for reverse causality. For instance, mental health challenges may also influence factors such as employment status or social support, rather than solely resulting from them. Other limitations include reliance on self-reported data that may introduce bias, exclusion of eight districts in Cabo Delgado due to security issues affecting representativeness, a narrow focus on major depressive disorder and generalized anxiety disorder, and the omission of contextual factors like exposure to violence or the impact of COVID-19. In addition, this study relies on secondary data from the 2022–23 Demographic Health Survey (DHS) in Mozambique. Consequently, we were unable to modify or clarify survey questions, including the employment type or help-seeking question which was phrased as “Ever tried to seek help for the things you experienced.” The wording is somewhat general and may capture help-seeking beyond mental health issues. As a result, these rates should be interpreted with caution.

However, the study's strengths are notable: it uses a large, nationally representative sample providing comprehensive insights, employs recent data from 2022–23, utilizes validated assessment tools (PHQ-9 and GAD-7) for the Mozambican population, includes a thorough analysis of sociodemographic factors, focuses on a critical age group (15–24 years), considers help-seeking behaviors, and applies rigorous statistical methods to ensure national representativeness and significance of findings.

## Conclusion

The findings highlight significant mental health challenges within this demographic, with notable variations across different sociodemographic groups. Females, those not currently working, and individuals from poorer households exhibited higher prevalence rates of MDD and GAD. Additionally, regional disparities were evident, with certain provinces showing significantly higher odds of these mental health disorders. The study also underscores the low rates of help-seeking behaviors among youth experiencing mental health issues, pointing to potential barriers such as stigma and lack of access to mental health services. These insights are crucial for informing targeted interventions and policy decisions aimed at improving mental health outcomes for young people in Mozambique. By addressing the identified sociodemographic drivers and enhancing mental health care accessibility, it is possible to mitigate the long-term impacts of these disorders on the youth population.

## Data Availability

The Mozambique DHS 2022–23 is a publicly available dataset and could be downloaded through the WHO official website (https://dhsprogram.com/data/available-datasets.cfm) upon a reasonable request by a registered user.
